# Liver transplant assessment for hepatocellular carcinoma: a single-centre experience

**DOI:** 10.1136/flgastro-2024-102773

**Published:** 2025-02-10

**Authors:** Rosemary Elizabeth Faulkes, Sean Morris, Oliwia Bolimowska, Zaira Rehman, Nadir Abbas, Bobby V M Dasari, Neil Rajoriya, Tahir Shah, Shishir Shetty

**Affiliations:** 1 Institute of Immunology and Immunotherapy, University of Birmingham, Birmingham, UK; 2 Liver Unit, University Hospitals Birmingham NHS Foundation Trust, Birmingham, UK; 3 National Institute of Health Research Birmingham Biomedical Research Unit, Birmingham, UK

**Keywords:** HEPATOCELLULAR CARCINOMA, LIVER TRANSPLANTATION, CANCER EPIDEMIOLOGY

## Abstract

**Objective:**

The incidence of hepatocellular carcinoma (HCC) continues to rise dramatically in the UK. Liver transplantation offers a potential cure and there is a large body of evidence demonstrating good outcomes. However, there is a paucity of data on the assessment, acceptance rates, and reasons for turning down liver transplantation in HCC.

**Methods:**

We undertook an analysis of all patients with HCC referred for liver transplant assessment to a tertiary liver centre between January 2015 and January 2020. Patient and tumour demographics, assessment outcomes and overall survival were analysed. Multivariate analysis was performed on factors affecting listing decisions. To evaluate the impact of the COVID-19 pandemic, data collection was extended from March 2020 to March 2021.

**Results:**

Of 263 patients with HCC who completed liver transplant assessment, 168 (64%) were accepted for listing. The most common factors associated with a decision not to list a patient were medical comorbidities (n=50, 56.2% of those not listed) and rapid tumour progression (n=25, 26.3%). Of patients who were listed, 145 (86.4%) received a liver transplant. Five year survival from the time of transplant assessment was 68% with transplant and 12% without.

The pandemic resulted in more patients progressing out of criteria after listing. Prepandemic median dropout per annum was 2% (0%–9%), compared with 25% during the pandemic study period.

**Conclusion:**

This study provides outcomes on patients with HCC referred for transplant assessment, identifying factors for non-listing and confirming the negative impact of decreased transplant activity during the pandemic on waiting list dropouts for HCC patients.

**WHAT IS ALREADY KNOWN ON THIS TOPIC**

- Hepatocellular carcinoma (HCC) is the third-leading cause of cancer death worldwide.
- Liver transplantation offers the best prognosis for localised disease; up to 25% of liver transplants in the UK are performed to treat HCC.
- Transplant assessment is an in-depth, multidisciplinary process.

**WHAT THIS STUDY ADDS**

- Outcomes on patients with HCC referred for transplant assessment, identifying acceptance rates and factors for non-listing.
- Confirms the negative impact of decreased transplant activity during the COVID-19 pandemic on waiting list dropouts for HCC patients.

**HOW THIS STUDY MIGHT AFFECT RESEARCH, PRACTICE OR POLICY**

- Improve counselling for patients with HCC referred for liver transplant assessment.

## Introduction

 Hepatocellular carcinoma (HCC) is the third-leading cause of cancer-related death worldwide.^1^ Over 90% of cases arise in people with underlying liver fibrosis or cirrhosis. The incidence of chronic liver disease (CLD) is increasing, particularly in Europe and North America, which directly affects liver cancer epidemiology.^2^ In the UK, net cancer mortality fell by 10% over 12 years, however, liver cancer deaths rose by 45% in the same period.^3 4^

CLD not only drives tumour formation but also makes cancer management more challenging. Liver dysfunction and portal hypertension may render ablation or resection too high risk.^5^ The best prognosis for localised, early-stage HCC is achieved through liver transplantation. Inclusion criteria and assessment of transplant benefits for HCC continue to be refined as understanding of tumour biology and organ utility evolves.^6^ Nevertheless, HCC is the primary indication for up to a quarter of liver transplants performed in the UK.^7^

Patient selection for liver transplantation requires a comprehensive, multidisciplinary assessment of general fitness, frailty and medical comorbidities. Engagement with the medical team and commitment to long-term follow-up are important, taking into consideration the patient’s social support, risk of alcohol relapse and health beliefs.^7^ In the UK, transplantation for HCC is performed according to nationally agreed parameters based on Milan criteria.^8^ Patients beyond criteria may be assessed for transplant after downstaging as part of a service evaluation, whereby the HCC is controlled for 6 months following treatment and Duvoux score is ≤2.^9^

The study aims were to evaluate rates and the impact of COVID-19 pandemic on transplant assessment and outcomes for patients with HCC at a single institution.

## Methods

Data were collated from electronic records and the liver transplant database at the Queen Elizabeth Hospital, Birmingham. Patients aged >16 referred for transplant assessment between January 2015 and January 2020 with HCC as primary indication were included. Cancer diagnosis was based on radiological findings and/or lesional biopsy. All patients were discussed in the hepatobiliary multidisciplinary meeting. Patients with incidental HCC on explant were excluded.

Data were collected regarding patient demographics, liver disease, comorbidities, tumour characteristics, use of bridging and downstaging therapies. Diagnosis of cirrhosis was based on a combination of biochemical and radiological signs of synthetic dysfunction, portal hypertension and irregular liver edge. Time from referral to presentation at listing meeting, date of transplant and date of last follow-up or death were documented. Where applicable, reasons for incomplete assessment or unsuitability for listing were recorded. In some cases, a clinical diagnosis of frailty was made at the listing meeting following the presentation of specialist opinions from the transplant assessment team.

Index of multiple deprivation deciles was calculated for patients in England using an online government resource (2019) based on patient postcode.^10^ The Welsh index of multiple deprivation is calculated differently; therefore, it was not used in this analysis as results are not directly comparable with English data.^11^

To evaluate the impact of the COVID-19 pandemic on transplant assessment, listing and outcomes for HCC at our institution, the number of patients referred, assessed and listed for transplant between March 2020 and March 2021 were also recorded. Details regarding bridging therapy and progression beyond the listing criteria were noted.

Patients and the public were not involved.

Data analysis was performed using GraphPad Prism (V.10.3.1) and SPSS (IBM, V.29.0.1.1). Normally distributed data were recorded as mean and SD. Non-normally distributed data were recorded as median and IQR. χ^2^ test was used for analysis of proportions. Significance was calculated using unpaired t-test or Mann-Whitney U test. Univariate analysis was performed regarding decision to list for transplant. Statistically significant factors were included in multivariate analysis. Survival data were assessed using log rank (Mantel-Cox) test. P values <0.05 were deemed statistically significant.

## Results

### Patient demographics

**Figure 1:**
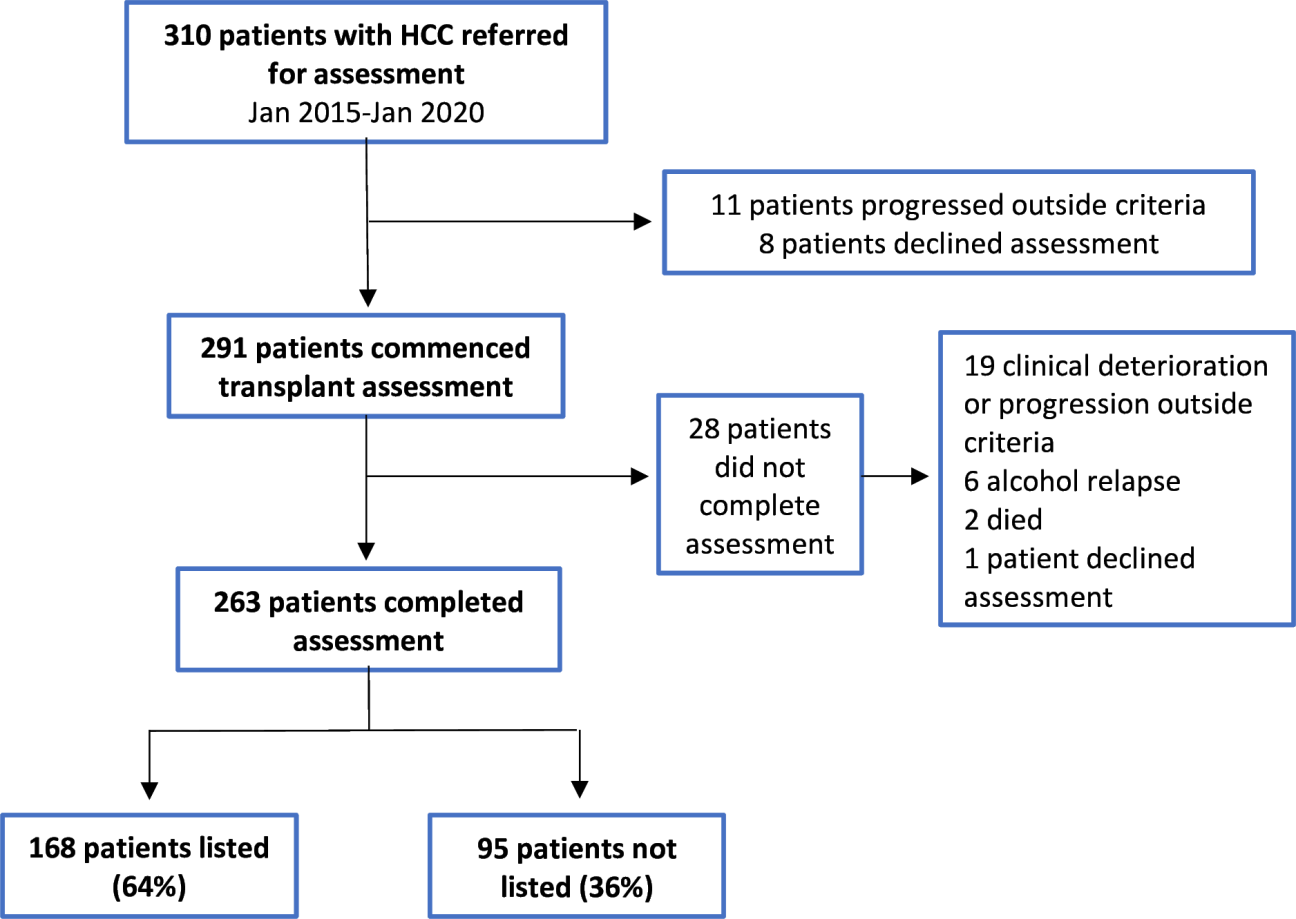
Flow diagram of study selection criteria and outcomes for patients with HCC referred for transplant assessment. HCC, hepatocellular carcinoma.

310 patients with HCC were referred for liver transplant assessment between January 2015 and January 2020, of whom 19 (6.1%) did not commence assessment due to progression outside HCC listing criteria (n=11, 3.5%) or patient choice (n=8, 2.6%) (figure 1). Within the remaining cohort of 291 patients, 28 (9%) commenced but did not complete transplant assessment due to clinical deterioration or progression outside criteria (n=19, 6.5%), alcohol relapse (n=6, 2%), death (n=2, 0.7%) or patient choice (n=1, 0.3%). In total, 90% (n=263) of assessments were completed, of which 168 (64%) patients were listed. Patient and tumour demographics are shown in table 1.

**Table 1:** Patient and tumour demographics of all patients with HCC assessed for liver transplantation January 2015–January 2020

Demographics	All patients n=263	Listed n=168	Not listed n=95	P value
Male	210 (80%)	136 (81%)	74 (78%)	0.56
Ethnicity				
Asian	19 (7%)	11 (7%)	8 (8%)	0.77
Black	5 (2%)	3 (2%)	2 (2%)	>0.99
Chinese	2 (1%)	2 (1%)	0	0.54
Mixed	4 (2%)	4 (2%)	0	0.3
Not specified	56 (21%)	23 (14%)	33 (35%)	<0.05
Other	3 (1%)	2 (1%)	1 (1%)	>0.99
White	174 (66%)	123 (73%)	51 (54%)	<0.05
Age (years) (IQR)	62 (56–66)	61 (55–65)	64 (58–67)	<0.05
Cirrhosis present	250 (95%)	156 (93%)	94 (99%)	
Aetiology of cirrhosis				
ArLD	64 (24%)	35 (21%)	29 (31%)	0.32
MASLD	55 (21%)	32 (19%)	23 (24%)	0.12
Viral	64 (24%)	46 (27%)	18 (19%)	<0.05
Mixed pathology	51 (19%)	32(19%)	19 (20%)	<0.05
HH	7 (3%)	6 (4%)	1 (1%)	<0.05
PBC	9 (3%)	7 (4%)	2 (2%)	0.08
Other	13 (5%)	10 (6%)	3 (3%)	<0.05
Tumour size (cm) (SD)	2.9 (1.15)	2.9 (1)	3.1 (1.26)	0.36
Number of tumours (SD)	1.5 (0.99)	1 (0.9)	2 (1.1)	<0.05
AFP (kU/L) (IQR)	6 (3–19)	6 (3–16)	6 (4–25)	0.51
UKELD (IQR)	48 (46–51)	48 (46–51)	49 (47–52)	0.07
Diabetes	110 (42%)	58 (34%)	52 (55%)	<0.05
IHD	17 (6%)	7 (4%)	10 (11%)	<0.05

AFP, alpha foetoprotein; ArLD, alcohol-related liver disease; HCC, hepatocellular carcinoma; HH, hereditary haemochromatosis; IHD, ischaemic heart disease; MASLD, metabolic-associated steatotic liver disease; PBC, primary biliary cholangitis; UKELD, United Kingdom Model for End-Stage Liver Disease.

Of completed assessments, the greatest number of referrals were within the West Midlands (n=59, 22%), followed by Merseyside (n=29, 11%), Shropshire (n=18, 6.8%) and Cheshire (n=17, 6.5%) (online supplemental table 1).

### Transplant assessment outcomes

Of the 95 patients who were not listed, the most common reason was being too high risk for transplant due to comorbidities or frailty (n=50, 52.6%), followed by cancer progression beyond transplant criteria (n=25, 26.3%), patient choice (n=11, 11.6%), no active cancer (n=5, 5.3%) and risk of alcohol relapse (n=4, 4.2%). One patient required veno-venous bypass for transplant and was transferred to another centre with capabilities to perform this.

Rates of listing varied according to underlying liver disease; 71.9% of patients with viral hepatitis were listed, compared with 54.7% with alcohol-related liver disease (ArLD) and 54.5% with metabolic-associated steatotic liver disease (MASLD). Those with viral hepatitis were younger at time of transplant assessment compared with ArLD and MASLD (median ages 58, 63 and 63 respectively).

In univariate analysis, age, deprivation index, diabetes, tumour size and tumour number were significantly associated with being listed. After multivariate analysis of these factors, patients were significantly less likely to be listed if they had larger tumours (OR 0.59, p<0.001) or multiple tumours (OR 0.62, p=0.013) (table 2). Sex, ischaemic heart disease (IHD), alpha foetoprotein (AFP) and United Kingdom Model for End-Stage Liver Disease (UKELD) did not influence listing on univariate analysis. Regarding ethnicity, 66% of the patients assessed were white. As 21% did not have ethnicity classified, the remaining cohort was too small to permit meaningful analysis.

**Table 2:** Multivariate analysis of likelihood of listing for transplant according to patient and tumour factors

Demographics	OR (95% CI)	P value
Age	0.95 (0.91 to 1.00)	0.068
Deprivation Index	1.10 (0.98 to 1.23)	0.093
Diabetes	0.55 (0.29 to 1.06)	0.075
Tumour size	0.58 (0.43 to 0.80)	<0.001
Tumour number	0.62 (0.43 to 0.90)	0.013

Deprivation Index data were only available for 143 (54%) of the total cohort.

The deprivation index categorises areas with the most deprivation in the 1st decile and least deprivation in the 10th. Patients from deciles 1 and 2 had significantly higher median UKELD compared with deciles 9 and 10 (48 vs 46, p=<0.05) and a higher incidence of type II diabetes mellitus (44% vs 30%, p=0.26) and IHD (13% vs 9%, p=0.62) although the difference was not statistically significant.

### Bridging therapy

Between 53% and 71% patients/year received bridging therapy with transarterial chemoembolisation (TACE) (n=101, 38%), radiofrequency ablation (RFA) (n=42, 16%) and sterotactic ablative radiotherapy (SABR) (n=10, 4%). Some patients received more than one modality. One patient was bridged with selective internal radiation therapy and one with tyrosine kinase inhibitors (TKIs), both in addition to other therapies.

### Patient survival

Median time from diagnosis to discussion at the listing meeting was 5 months (IQR 3–8). Median wait time from listing to transplant was 2 months (IQR 0.9–4.2). Of 168 patients who were accepted on the waiting list, 145 (86%) received a liver transplant, 12 (7%) died prior to transplant, 4 (2%) removed due to clinical deterioration, 3 (1.7%) removed due to tumour progression, 3 (1.7%) removed at patient request and 1 (0.6%) transferred to another centre due to COVID-19 pressures.

**Figure 2:**
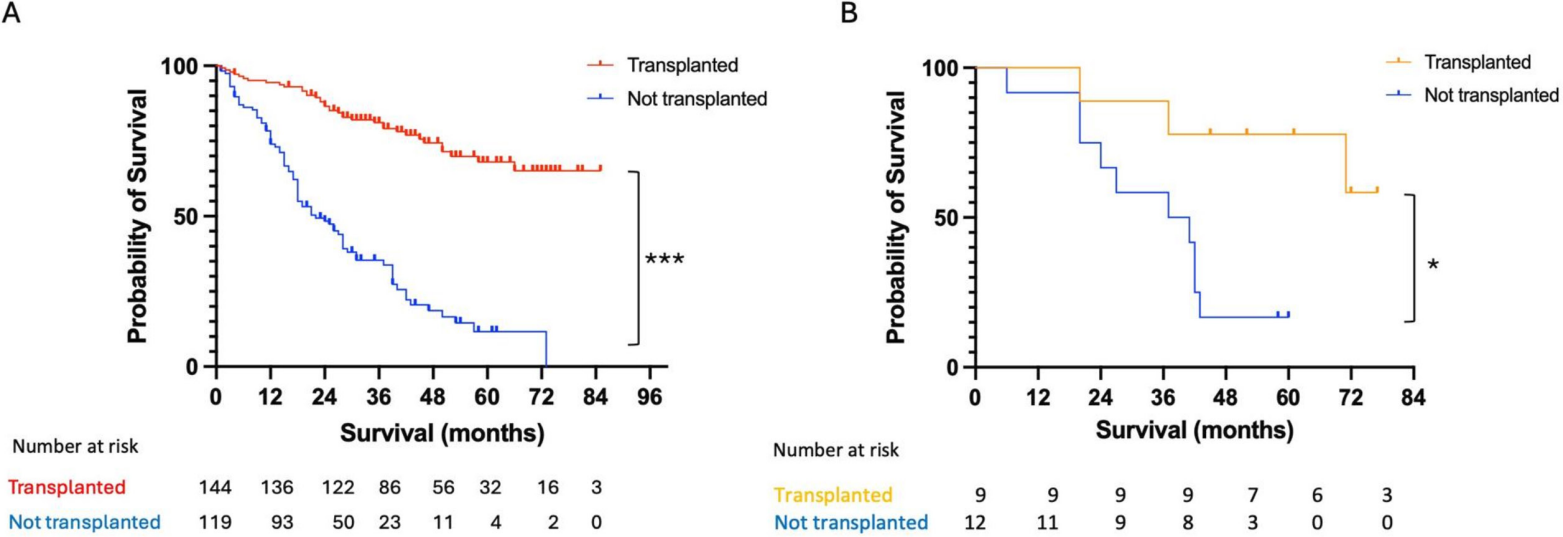
Overall survival of all patients with HCC assessed for liver transplant over a 5-year period, all-cause mortality from time of discussion at listing meeting. (**A**) All patients who completed liver transplant assessment between January 2015 and January 2020, n=263. (**B**) Patients assessed following successful downstaging, n=21. *p<0.05, ***p<0.0001 log rank (Mantel-Cox) test. HCC, hepatocellular carcinoma.

Overall survival at 1, 3 and 5 years from time of assessment was 95.0%, 81.1% and 68.0% for transplant recipients, and 73.4%, 35.4% and 11.6% without liver transplant. By 7 years, transplant-free survival was 0, whereas survival with liver transplant was 65.1% (figure 2).

### Downstaging

Between 2015 and 2021, 21 patients (7.2%) were successfully treated with TACE, SABR, ablation or TKI’s within UK downstaging criteria. Of these patients, 8 (38.1%) deteriorated due to liver decompensation or frailty and were unsuitable for listing, and 1 (4.8%) opted not to proceed with assessment. Of the remaining 12 patients who were downstaged and listed, 9 (42.9%) received a liver transplant, 2 (9.5%) progressed beyond transplant criteria and 1 patient (4.8%) died.

Overall survival at 1, 3 and 5 years without transplant was 91.6%, 66.6% and 16.6%, respectively, compared with 100%, 100% and 77.7% with transplantation (figure 2).

### Transplant assessment during the COVID-19 pandemic

Fewer patients were assessed between March 2020 and March 2021 than the preceding year (35 vs 74, respectively; online supplemental table 2). Median time from diagnosis to presentation at the listing meeting was 6.5 months (IQR 3–10). While median time for patients with HCC to receive a liver graft was 1 month during the pandemic, compared with 2 months in 2015–2020, overall fewer transplants were performed during this period (149 compared with average of 213/year between 2015 and 2020, all indications). Therefore, median time for patients with HCC on the waiting list until transplant, removal or death, was 4 months (IQR 1–11.5), double the 2-month average in 2015–2020 (IQR 1–6).

The proportion of patients removed from the waiting list due to HCC progression also increased during the pandemic. During 2015–2020, between 0% and 9% (n=0–7) of listed patients progressed out of criteria per year, compared with 25% (n=6) in March 2020–March 2021 (online supplemental table 2).

## Discussion

This study provides a detailed analysis of transplant assessment in patients with HCC, demonstrating that tumour size and number significantly affected listing (p≤0.05) but age, sex, deprivation score, UKELD and AFP did not. As HCC was the primary indication for transplant rather than CLD, UKELD is less likely to influence listing decisions in these cases. AFP is part of the inclusion criteria for transplantation, therefore, patients with high AFP may not have been referred for assessment and thus not included in our analysis. Indeed, HCC presents a unique cohort in transplant assessment, as management and risk assessment of cancer must be performed in parallel to evaluation of transplant-associated risks. To date, the authors are not aware of other reports of HCC transplant assessment in the literature.

The majority of patients in this dataset were male and aged over 50, consistent with the known epidemiology of HCC.^12^ A higher proportion of patients with viral hepatitis were listed (p<0.05), whereas there was no difference for ArLD, MASLD or primary biliary cholangitis. Those with viral hepatitis were younger at time of assessment compared with ArLD and MASLD. This may reflect differences in tumour biology according to aetiology of underlying liver disease. Viral-HCC is often diagnosed at an earlier age than MASLD and biliary disease.^13 14^ Patients with viral hepatitis may represent a fitter cohort with few or no comorbidities compared with other CLD groups, and therefore, more often considered suitable candidates for transplantation.

Over one-third (n=95, 36%) of those who completed assessment were not added to the waiting list, primarily due to comorbidities and/or frailty. The multidisciplinary team manage frailty through diet, exercise and medicines optimisation, such as treating encephalopathy. At our centre, physiotherapists deliver a bespoke prehabilitation programme for frail patients prior to assessment and transplant. However, this service is not widely available. Further development of liver services nationally is required to improve access to specialist dieticians and physiotherapists to improve quality of life and survival for patients with CLD and HCC.

While deprivation was not associated with listing outcome, analysis was affected by limitations of the national database, as only 54.4% of assessed patients were included. From the data available, patients from areas with higher deprivation had more advanced liver disease, as indicated by higher median UKELD. Premature mortality due to liver disease, including but not limited to HCC, is almost four times higher in the most deprived areas of England compared with the least (41.0 per 100 000 vs 10.5 per 100 000).^15^ Degree of deprivation correlates with HCC incidence.^12 16^ This study reflects widening health inequalities between the most and least deprived populations in England and Wales over the past two decades.^16 17^ A recent review has highlighted disparities in access to HCC surveillance, diagnosis and treatment in Europe, and increased incidence of HCC in low-income households within areas of high deprivation.^18^ Efforts to redress these inequalities are being made through local strategies, such as developing community outreach teams to increase cirrhosis diagnosis and enrolment in HCC surveillance. However, broader improvements require policy change and investment at a public health level.

While frailty and tumour burden are anticipated risk factors against transplantation, this study shows the third leading reason not to list was patient choice. In comparison, a Canadian study of 337 patients assessed for transplant for all indications reported 43.9% were turned down for listing, but patient decision was not cited as a reason.^19^ We hypothesise that the absence of symptoms for some patients with HCC may make them reluctant to undergoing major surgery. Further exploration is needed using a patient and public involvement research approach.

Five year survival with transplantation was comparable to published rates of 68%–78%.^20^,^22^ Transplant-free survival was significantly reduced, likely due to multiple factors including aggressive tumour biology and life-limiting comorbidities; nevertheless, it highlights the natural course of HCC and CLD, and the limited survival benefit of alternate treatments in advanced HCC. Future work is required to discover whether immunotherapy and other novel systemic therapies will translate to improved outcomes in instances where transplantation is not possible.

The onset of the COVID-19 pandemic necessitated a shift in treatment to facilitate transplantation for HCC. Using outpatient radiotherapy rather than RFA or TACE enabled consistent delivery of bridging therapy during the first two waves of the pandemic, with comparable survival rates between treatments.^23^,^25^ The Birmingham transplant unit also modified intensive care strategy to enable transplantation for urgent cases, including advanced HCC, despite a 200% increase in ventilated patients during the first wave.^26^ However, the inevitable fall in transplant activity across the UK prolonged waiting times during the pandemic, resulting in a greater rate of progression beyond criteria compared with the previous 5 years. While bridging therapy may buy time for some patients, that time is finite.

A 5-year post-transplant survival following downstaging was longer than the general cohort, although within a small sample (n=9). A multicentre US study reported 5-year survival of 67.9% in patients downstaged to within Milan criteria.^22^ The downstaged cohort has demonstrated favourable tumour biology by prolonged response to bridging therapy, which can be a positive predictive factor in post-transplant survival.^27^ As more effective systemic and combined therapies become available, the number of patients downstaged to within acceptable criteria will likely increase. The future development of UK HCC services will need to take this into account, such as creating a clinic dedicated to a downstaging pathway.

This study was limited by being a retrospective, single-centre study. Classification of deprivation deciles was not available for many patients in England due to gaps in the national database, and the different classification system in Wales prevented inclusion of Welsh data. A further limitation is the lack of unified patient records, such that information from referring hospitals was not accessible unless specified in the referral letter.

In conclusion, this study produced essential data to inform discussions with patients with HCC who are referred for liver transplant assessment, and to support regional referral centres in counselling patients regarding acceptance rates and overall outcomes. Our data clearly demonstrate that survival for patients unsuitable for liver transplantation is poor and thus, in such cases, healthcare professionals should consider palliative support and discussions regarding advanced care planning. Evaluation during the COVID-19 pandemic highlighted that, despite consistent delivery of bridging therapy, reduced transplant activity increased the waiting list dropout rate. This study highlights the importance of developing resources to aid transplantation in a timely manner.

## Supplementary material

10.1136/flgastro-2024-102773 online supplemental file 1

10.1136/flgastro-2024-102773 online supplemental file 2

## Data Availability

All data relevant to the study are included in the article or uploaded as supplementary information.
