# Supplementary material for: Liver transplant assessment for hepatocellular carcinoma: a single-centre experience
Source: Frontline Gastroenterol. 2025 Feb 10;16(5):e102773. doi: 10.1136/flgastro-2024-102773 (PMC12418541; doi:10.1136/flgastro-2024-102773)
Supplement: online supplemental file 1 [file flgastro-16-5-s001.pdf]

Supplementary materials

**Region**	**Referrals / million population per region**
Shropshire	36
West Midlands	24
Merseyside	21
Worcestershire	18
Cheshire	15
Staffordshire	15
Mid and South West Wales	14
Warwickshire	14
Leicestershire	13
Northamptonshire	11
South East Wales	9
Berkshire	9
North Wales	9
Derbyshire	7
Gloucestershire	6
Oxfordshire	6
Lancashire	3
Greater Manchester	1

SP Table 1 Number of HCC referrals for liver transplant assessment by region, 2015-2020

**Date of assessment**	**N assessed**	**N listed**	**N transplanted**	**N progressed out of criteria on waiting list**
2015	31	25	25 (100%)	0 (0%)
2016	48	31	28 (90%)	1 (2%)
2017	36	20	19 (95%)	0 (0%)
2018	74	51	42 (82%)	7 (9%)
2019-Jan 2020	74	42	30 (71%)	1 (2%)
Feb 20-March 21	35	24	16 (66%)	6 (25%)

SP Table 2 Transplant assessment outcomes for patients with HCC by year, 2015-2021.
