# Supplementary material for: Liver transplant assessment for hepatocellular carcinoma: a single-centre experience
Source: Frontline Gastroenterol. 2025 Feb 10;16(5):e102773. doi: 10.1136/flgastro-2024-102773 (PMC12418541; doi:10.1136/flgastro-2024-102773)
Supplement: online supplemental file 2 [file flgastro-16-5-s002.pptx]

## Slide 1
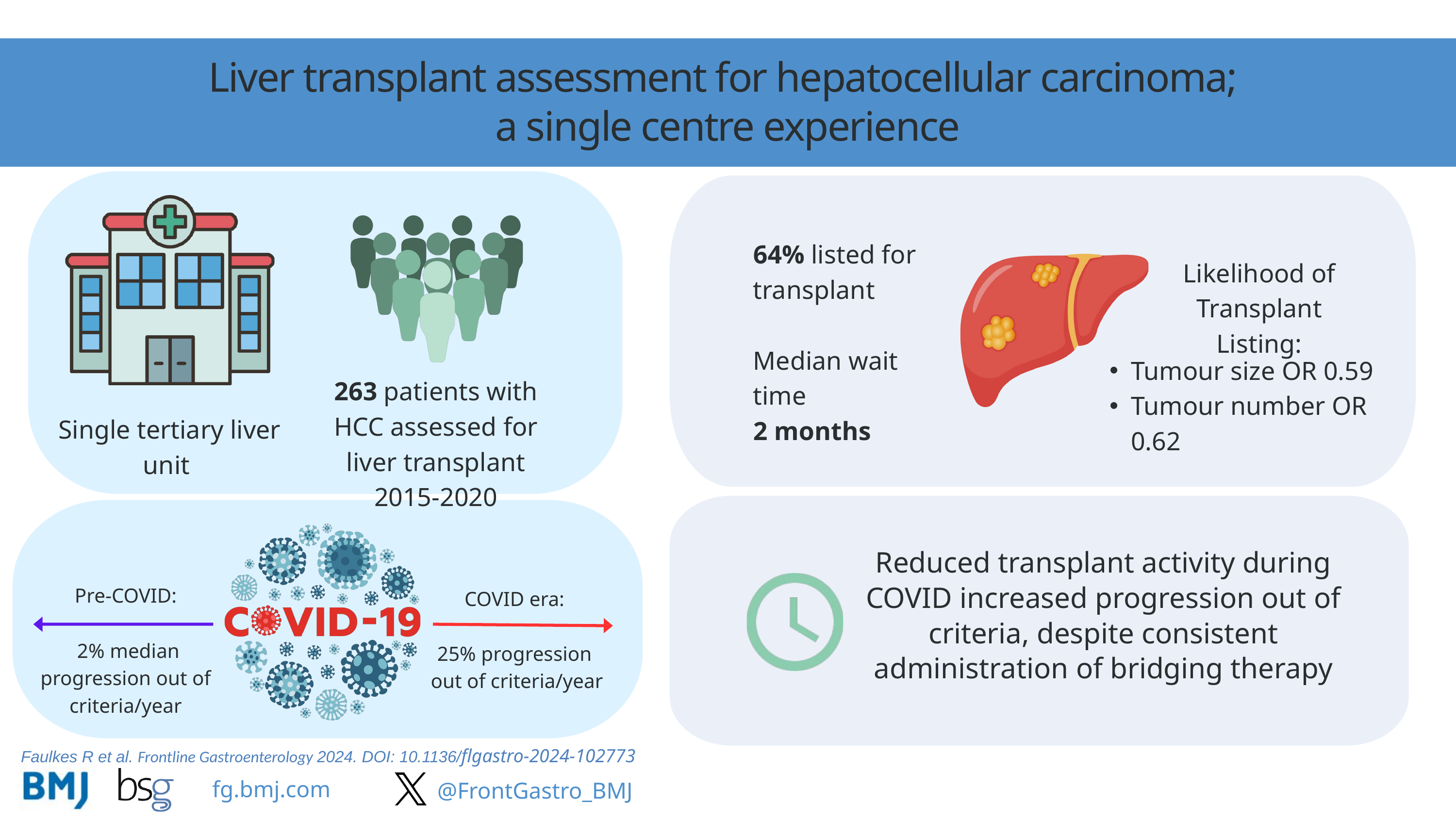

Liver transplant assessment for hepatocellular carcinoma;
a single centre experience
64% listed for transplant
Median wait time
2 months
Likelihood of Transplant Listing:
Tumour size OR 0.59
Tumour number OR 0.62
263 patients with HCC assessed for liver transplant 2015-2020
Single tertiary liver unit
Reduced transplant activity during COVID increased progression out of criteria, despite consistent administration of bridging therapy
Pre-COVID:
 2% median progression out of criteria/year
COVID era:
25% progression
out of criteria/year
Faulkes R et al. Frontline Gastroenterology 2024. DOI: 10.1136/flgastro-2024-102773
fg.bmj.com
@FrontGastro_BMJ
